# Supplementary material for: Failed Disruption of Tick Feeding, Viability, and Molting after Immunization of Mice and Sheep with Recombinant Ixodes ricinus Salivary Proteins IrSPI and IrLip1
Source: Vaccines (Basel). 2020 Aug 26;8(3):475. doi: 10.3390/vaccines8030475 (PMC7564719; doi:10.3390/vaccines8030475)
Supplement: Supplementary file 1 [file vaccines-08-00475-s001.pdf]

vaccines

MDPI

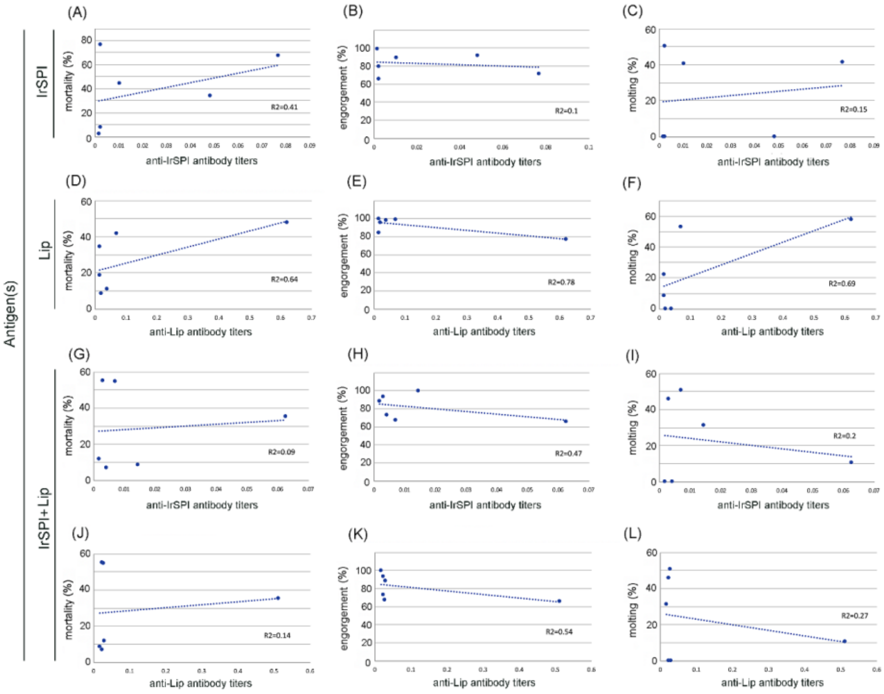

Figure 1. Correlation between antibody titers against recombinant IrSPI (**A–C, G–I**) and IrLip1 (D–F, **J–L**) in sheep vaccinated against IrSPI (**A–C**), IrLip1 (**D–F**), both IrSPI and IrLip1 (**G–L**), and effect on mortality, engorgement and molting of *I. ricinus* larvae at Day 45 after sheep immunization. The linear correlation coefficients (R2) are shown (*N* = 6). Antibody titers are represented as arbitrary units.
